# Exploring the Mechanism of Edaravone for Oxidative Stress in Rats with Cerebral Infarction Based on Quantitative Proteomics Technology

**DOI:** 10.1155/2022/8653697

**Published:** 2022-01-04

**Authors:** Guozuo Wang, Xiaomei Zeng, Shengqiang Gong, Shanshan Wang, Anqi Ge, Wenlong Liu, Jinwen Ge, Qi He

**Affiliations:** ^1^Hunan University of Chinese Medicine, Changsha, Hunan, China; ^2^People's Hospital of Ningxiang City, Ningxiang, Hunan, China; ^3^The First Affiliated Hospital of Hunan University of Chinese Medicine, Changsha, Hunan, China

## Abstract

**Objective:**

To explore the mechanism of edaravone in the treatment of oxidative stress in rats with cerebral infarction based on quantitative proteomics technology.

**Method:**

The modified Zea Longa intracavitary suture blocking method was utilized to make rat CI model. After modeling, the rat was intragastrically given edaravone for 7 days, once a day. After the 7-day intervention, the total proteins of serum were extracted. After proteomics analysis, the differentially expressed proteins are analyzed by bioinformatics. Then chemoinformatics methods were used to explore the biomolecular network of edaravone intervention in CI.

**Result:**

The neurological scores and pathological changes of rats were improved after the intervention of edaravone. Proteomics analysis showed that in the model/sham operation group, 90 proteins in comparison group were upregulated, and 26 proteins were downregulated. In the edaravone/model group, 21 proteins were upregulated, and 41 proteins were downregulated. Bioinformatics analysis and chemoinformatics analysis also show that edaravone is related to platelet activation and aggregation, oxidative stress, intercellular adhesion, glycolysis and gluconeogenesis, iron metabolism, hypoxia, inflammatory chemokines, their mediated signal transduction, and so on.

**Conclusion:**

The therapeutic mechanism of edaravone in the treatment of CI may involve platelet activation and aggregation, oxidative stress, intercellular adhesion, glycolysis and gluconeogenesis, iron metabolism, hypoxia, and so on. This study revealed the serum protein profile of edaravone in the treatment of cerebral infarction rats through serum TMT proteomics and discovered the relevant mechanism of edaravone regulating iron metabolism in cerebral infarction, which provides new ideas for the study of edaravone intervention in cerebral infarction and also provides reference information for future research on the mechanism of edaravone intervention in iron metabolism-related diseases.

## 1. Introduction

Stroke is the second most common cause of death in the world, and it is also the main cause of adult disability [1]. As the American Heart Association and the National Institute of Neurological Diseases and Stroke reported, about 795,000 Americans suffer from ischemic stroke (such as cerebral infarction, CI) each year, and 220,000 die from ischemic stroke each year [2, 3]. The “China Cardiovascular Disease Report 2018” compiled by the National Center for Cardiovascular Diseases showed that the prevalence of cardiovascular disease in China is on the rise. It is estimated that there are 290 million people suffering from cardiovascular disease, of which 13 million have stroke and 11 million have coronary heart disease [4]. According to the China Stroke Prevention Report (2015), about 15% of people over 40 years old are high-risk groups. From 2011 to 2013, the comprehensive standardized prevalence rate of ischemic stroke was about 2%, and the prevalence rate increased by 8.1% [5]. According to data from the World Bank, by 2030, China will have 31.77 million patients with ischemic stroke, which will cost US$40 billion each year [6]. Current research showed that oxidative stress plays an important role in the pathological process of ischemic brain injury. After cerebral ischemia, the production of superoxide anions, hydrogen peroxide, hydroxyl free radicals and peroxynitrite anions increases due to factors such as excitotoxicity, inflammatory response, and hypoxic environment inhibiting cell respiration, especially during reperfusion [7–9]. Hydroxyl free radicals and peroxynitrite anions can promote protein nitrification and oxidation, lipid peroxidation, mitochondrial and DNA damage, inflammatory activation, cell necrosis, and apoptosis, which can cause brain damage [10, 11].

As a scavenger of oxidative stress, edaravone is widely used in China and other Asian countries, but it has not been approved for use in Western countries [12]. A systematic review involving 49 animal experiments showed that the functional and cognitive prognosis of edaravone in an animal model of focal cerebral ischemia increased by 30.3% and 25.5%, respectively [13]. A systematic review published by Yang et al. included 18 randomized controlled trials involving 1802 patients, and the results showed that edaravone can significantly reduce the mortality or long-term disability rate of acute ischemic stroke (AIS) [14]. Both of these systematic reviews support the effectiveness of edaravone for acute ischemic stroke. Although the current research shows that edaravone can mainly improve the oxidative stress in CI [15], there is no systematic research on the mechanism of edaravone in the treatment of ischemic brain injury such as proteomics and chemoinformatics. In particular, with the rapid development and rapid iteration of current proteomics technology, the large amount of data obtained from proteomics detection and analysis represents all the processes and changes that occur in the cell [16, 17]. Therefore, in this research, proteomics and bioinformatics analysis strategies would be utilized to observe the changes of related indicators after edaravone in the treatment of CI and further explore the mechanism of edaravone to protect ischemic brain injury.

## 2. Material and Methods

### 2.1. Experimental Materials

SPF-grade male SD rats, weighing 200∼230 g, were provided by Laboratory Animal Technology Co., Ltd., and the certificate number is SCXK: (Xiang) 2017–0012 (edaravone injection, article number, company batch number 1511001Y). RIPA lysate was obtained from Thermo Inc.; iodoacetamide was obtained from IAM, Sigma Aldrich; trypsin was obtained from Promega; ammonium bicarbonate was obtained from Sigma Aldrich Inc.; SOD, MDA, and GSH-px kits were obtained from Shanghai Enzyme Linked Biotechnology Co., Ltd.; rat angiotensinogen (AGT) ELISA kit (E-EL-M0013c) was purchased from Elabscience Inc. The Catalase (CAT) kit was purchased from Nanjing Jiancheng Biotechnology Research Institute. Protein quantification kit, Exactive Plus mass spectrometer, EASY-n LC 1000 liquid analyzer, C18 analytical column, EASY-Spray Column, AcclaimPep Map 100 were obtained from Thermo Inc. Nano Vue UV-Visible Spectrophotometer was obtained from GE Healthcare. Laser Doppler flow meter was obtained from Mo or VMS-LDF, Moor Instruments, Aminster, Devon, UK.

### 2.2. Experimental Methods

#### 2.2.1. Animal Grouping and Intervention

Thirty male healthy SD rats were randomly divided into 3 groups by random number table method: sham operation group (CN group), CI model group (CI group), and edaravone group (CE group), 10 rats in each group. The rats in the model administration group were given edaravone by gavage with a dose of 3.5 g/kg. The sham operation group and the model group were given distilled water by gavage with a volume of 10 mL/kg. The drug was administered once a day at 14 : 00 for 7 consecutive days. All animals' care and experimental procedures were approved by the Animal Ethics Committee of Hunan University of Chinese Medicine and were in accordance with the National Institute of Health's Guide for the Care and Use of Laboratory Animals.

#### 2.2.2. Animal Modeling

After drug administration, according to the method of Longa et al. [18], the right middle cerebral artery occlusion (MCAO) model of rats was prepared by the suture method. First, the rats were anesthetized by intraperitoneal injection of 1% sodium pentobarbital (50 mg/kg), and then the right common carotid artery was carefully separated. Then, a 5–0 suture was used to insert a blunt nylon thread from the right external carotid artery of the rat. The thread enters the intracranial segment of the internal carotid artery from the side of the external carotid artery through the bifurcation of the common carotid artery and the extracranial segment of the internal carotid artery and reaches the branch of the middle cerebral artery for circular ligation. During the model preparation process of rats in the sham operation group, except that the nylon thread did not block the middle cerebral artery, the other operations were the same as those in the model group and edaravone group. After the rats were awake, the neurological function was scored according to Longa's 5-point system.

Zero points meant no neurological deficits. One point meant mild loss of nerve function: limited extension of the left forelimb, flexion when the tail is lifted. Two points meant moderate neurological deficit: rotating to the paralyzed side (left side) when crawling. Three points meant severe neurological deficit: falling into the paralyzed side (left side) while crawling. Four points meant difficulty in crawling and performance of decreased consciousness. Rats with a score of 1 to 3 were included in the experiment.

#### 2.2.3. Immunofluorescence Staining

After BrdU was dissolved in normal saline, the dose was determined at 100 mg/kg/d, and intraperitoneal injection was performed. The sections of brain tissue were immersed in 3% H2O2 deionized water for 10 min, washed with PBS for 5 min × 3 times, and immersed in 2 mol/L HCl at 37°C for 15 min. Then, the sections were washed with PBS for 5 min × 3 times; 5% goat serum was blocked at room temperature for 30 min, and the liquid was aspirated. After that, BrdU monoclonal antibody (1 : 100) of 10 *μ*L was added and incubated in 37°C water bath. Rhodamine (light emission wavelength 570 ∼ 590 nm, red light) staining was performed, 37°C water bath for 30 min. Finally, the slices were packaged with glycerin and observed with an OLYMPUS BX51 fluorescence microscope by the corresponding color filters at 520 and 580 nm, respectively. Rhodamine is red.

### 2.3. Protein Sample Processing

#### 2.3.1. Serum Protein and Brain Tissue Protein Extraction

After the rats were anesthetized with 1% pentobarbital sodium, blood was taken from the abdominal aorta, and the preserum was left standing. The rats were then sacrificed by cervical dislocation under anesthesia. Then, the brain tissue was taken out and washed with physiological saline, the brain tissue was put in the EP tube, RIPA lysis solution was added, and the brain tissue was cut into pieces with ophthalmological scissors and put it in a glass homogenizer for homogenization. The brain tissue homogenate was placed in a centrifuge at 4°C and centrifuged at 13,000 r/min for 30 minutes, and the precipitate was discarded. The upper liquid was the whole brain tissue protein.

#### 2.3.2. Enzymatic Hydrolysis of Protein

The protein sample was added with TCEP with a final concentration of 5 mmol/L, 37°C water bath for 30 minutes, and allowed to cool in room temperature. IAA with a final concentration of 10 mmol/L was added and kept in a 37°C dark water bath for 30 minutes. The enzyme solution was added to a water bath at a ratio of protein content to trypsin solution of 25 : 1 at 37°C overnight. On the second day, formic acid was added to the digested protein sample, and 0.1% was added to terminate the digestion reaction. The digested product was transferred to a 10 k Da ultrafiltration tube and centrifuged at 4°C at 10,000 r/min for 30 min. The protein in the lower layer of the ultrafiltration tube can be directly analyzed by mass spectrometry.

#### 2.3.3. TMT Labeling and High-Performance Liquid Chromatography (HPLC) Classification

The protein extract was used to remove the high-abundance proteins, and the BCA kit was used to determine the protein concentration. 20 *μ*g of the eluate was taken for SDS-PAGE electrophoresis to detect the removal of high-abundance proteins. Trypsin enzymatically hydrolyze peptides with Strata X C18 (Phenomenex) desalting and freeze-drying in vacuum. The peptide was dissolved with 0.5 M TEAB, and the peptide was labeled according to the TMT kit operating instructions. The peptides were fractionated by high pH reverse HPLC, and the column was Agilent 300Extend C18.

#### 2.3.4. LC-LTQ-MS/MS Analysis and Mass Spectrum Data Retrieval

In mass spectrometry analysis, the peptides were separated using the EASY-nLC 1000 ultra-high performance liquid system, and the Thermo ScientificTM Q ExactiveTM Plus was simultaneously used for detection and analysis. The peptides are separated and ionized and then enter the Q ExactiveTM Plus mass spectrometer for analysis. Peptide precursor ions and their secondary fragments are detected and analyzed by Orbitrap. The secondary mass spectrum data is retrieved by Maxquant (v1.5.2.8), and the mass spectrum quality control is performed at the same time.

### 2.4. Bioinformatics and Chemical Informatics Analysis

All the proteins retrieved from the database are analyzed, and the proteins whose expression changes are more than 1.3 times (fold change greater than 1.5 for upregulation and less than 0.67 for downregulation) are selected as differential proteins. Pubchem (https://pubchem.ncbi.nlm.nih.gov/) was used to retrieve the molecular structure of edaravone and saved as the “sdf” structure. Then, it was imported into Pharmmapper (http://lilab-ecust.cn/pharmmapper/) for potential target prediction [19]. The GeneCards database (https://www.genecards.org/) [20] and OMIM (https://omim.org/) [21] were used to retrieve genes related to CI and establish disease gene data sets.

The UniProt database is used to correct the names of differential proteins and official gene symbols (Table S1, see supplementary materials). David Ver 6.8 was used for gene ontology (GO) annotation analysis and functional clustering analysis of differential proteins, edaravone potential, and CI genes [22]. The online tool STRING (http://www.string-db.org) was used for protein interaction analysis of differential proteins [23].

### 2.5. Detection of Oxidative Stress Indicators in Brain Tissue

After the brain tissue was homogenized with physiological saline, the contents of SOD, MDA, GSH, and NO were determined according to the instructions of the kit.

### 2.6. Detection of Serum AGT and CAT by ELISA

The serum of each group of rats was collected, placed in an anticoagulation tube, shaken, centrifuged (3000 r/min) for 15 min, and the upper serum was collected. Serum AGT and CAT levels were detected by ELISA according to the instructions of the kit. Firstly, the AGT and CAT monoclonal antibodies are coated on the ELISA plate, and the standards and samples are added to make the AGT and CAT bound to the corresponding monoclonal antibodies. Biotinylated anti-rat AGT and CAT antibodies are added to form an immune complex and connect to the plate. Then, streptavidin labeled with horseradish peroxidase is combined with biotin, the enzyme substrate OPD is added, and after the yellow color appears, the stop solution sulfuric acid is added. The sample is detected by the microplate reader at a wavelength of 450 nm in accordance with the ELISA kit procedure. After the blank hole is zeroed, read the optical density (OD) value of each hole.

### 2.7. Statistical Analysis

The measurement data were expressed as mean ± standard deviation (SD). The neurological function score (mNSS score) of the rats was analyzed by one-way analysis of variance using SPSS software 19.0. *P* < 0.05 was considered to be statistically significant.

## 3. Results

### 3.1. Neurological Score and Pathological Changes

The neurological function score of each model group was in the range of 1–3 points, which can indicate to a certain extent that the model is successful. The neurological function scores of the CN group and the CE group were lower than those of the CI group, indicating that the neurological function after drug intervention was better than the cerebral infarction model without drug intervention and to a certain extent that the drug has an intervention effect on CI (Table 1).

**Figure 1:**
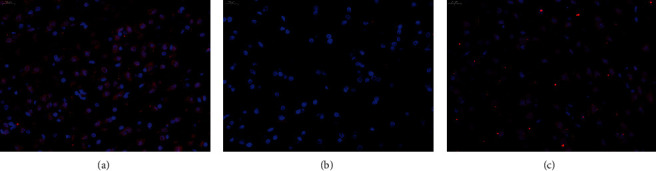
Pathological changes (400X). (a) Sham operation group. (b) Model group. (c) Edaravone group.

Under immunofluorescence, the BrdU signals appeared in the edaravone group, and it was considered that there were newborn nerve cells. The number of positive signals in the edaravone group was higher than that in the model group, indicating that the number of newborn nerve cells increased after drug treatment (Figure 1).

### 3.2. Proteomics Analysis Results

#### 3.2.1. Differential Expressed Protein

**Figure 2:**
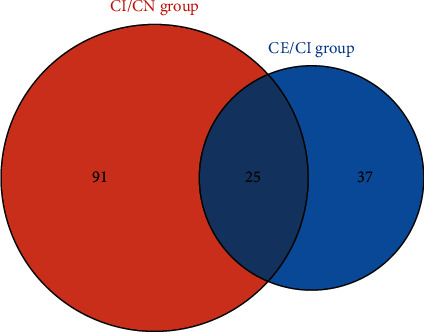
Venn diagram of CE/CI group and CI/CN group.

**Figure 3:**
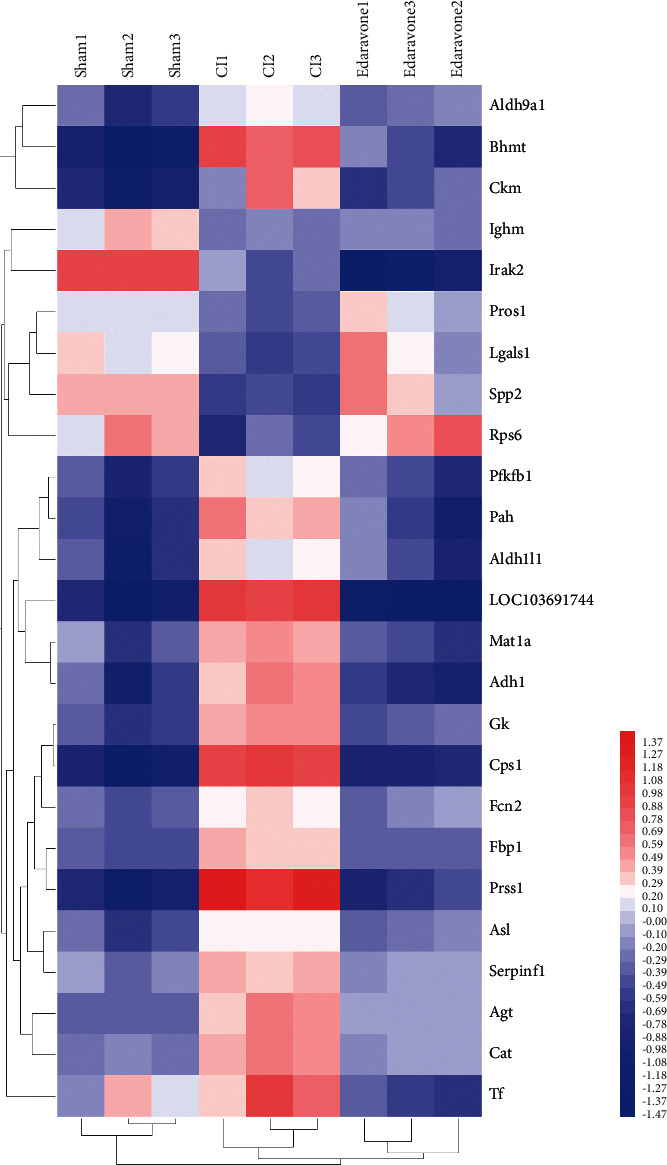
The expression matrix the 26 proteins.

A total of 1,340 proteins were identified in this study, of which 1,138 proteins contained quantitative information. With 1.3 times as the change threshold and *t*-test *P* value<0.05 as the standard, then among the quantified proteins, the expression of 90 proteins in the CI/CN comparison group was upregulated, and the expression of 26 proteins was downregulated (Table 2). In the CE/CI comparison group, the expression of 21 proteins was upregulated, and the expression of 41 proteins was downregulated (Table 3). The difference fold value change more than 1.5 times was regarded as a significant increase, and less than 0.77 was regarded as a significant decrease. There are overlapping proteins between CE/CI group and CI/CN group (Figure 2). They were considered to be the adjustable targets of edaravone after CI. The expression matrix of these proteins is shown in Figure 3.

#### 3.2.2. Proteomics Findings Validated by ELISA

**Figure 4:**
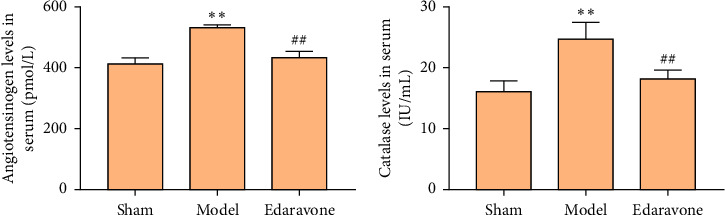
Serum AGT and CAT level (^∗∗^compared with sham operation group, *P* < 0.05; ^##^compared with model group, *P* < 0.05).

Compared with sham operation group, the AGT and CAT in model group were increased (*P* < 0.05). Compared with model group, the AGT and CAT in edaravone group were decreased (*P* < 0.05). This is consistent with the findings of proteomics (Figure 4).

#### 3.2.3. Bioinformatics Analysis

**Figure 5:**
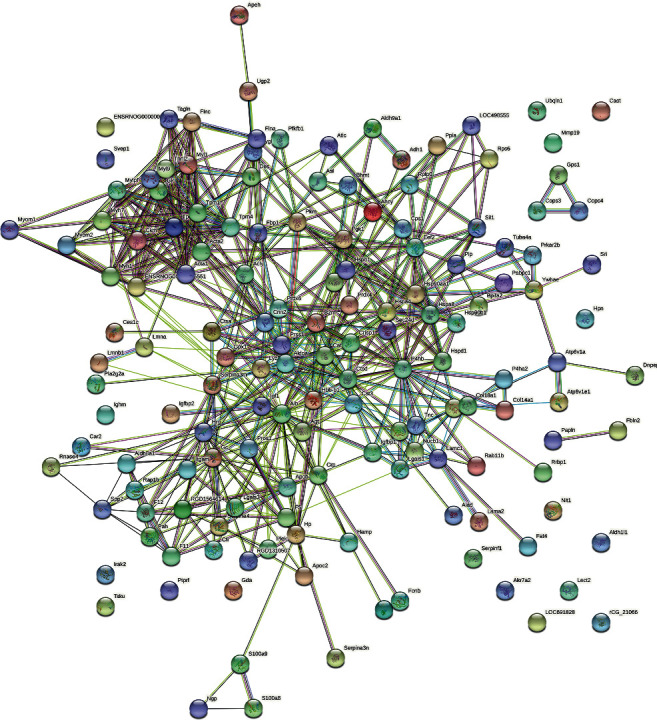
The PPI network of differentially expressed proteins.

**Figure 6:**
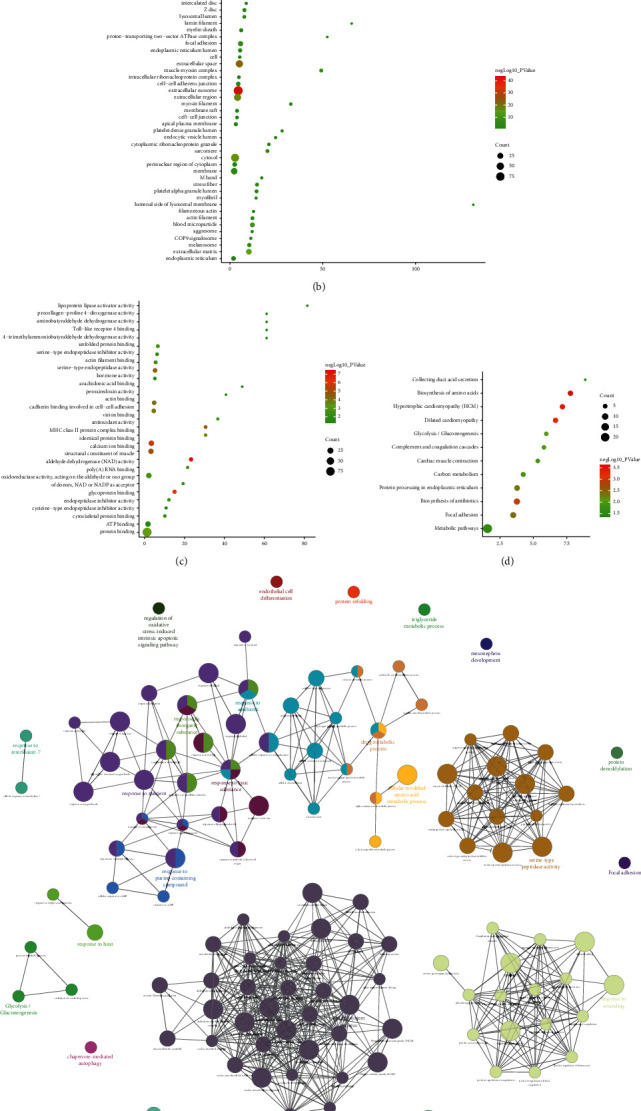
The results of enrichment analysis (a: biological processes; b: cell components; c molecular function; d signaling pathways; *X*-axis stands for fold enrichment. e and f Cluego analysis).

One hundred and fifty-three (153) differentially expressed proteins were introduced into String to construct PPI network (Figure 5) and subjected to enrichment analysis. The enrichment results show that these 153 differentially expressed proteins are related to 12 signaling pathways, 84 biological processes, 41 cell components, and 29 molecular functions. Their fold enrichment, *P* value and count of each signaling pathway, biological process, cell component, and molecular function are shown in Figure 6. The biological processes is mainly related to response to muscle filament sliding, platelet degranulation, muscle contraction, cellular oxidant detoxification, proteolysis, negative regulation of endopeptidase activity, response to drug, response to zinc ion, response to ethanol, protein refolding, retina homeostasis, response to unfolded protein, cell-cell adhesion, cardiac muscle contraction, response to hydrogen peroxide, skeletal muscle contraction, canonical glycolysis, defense response to fungus, response to selenium ion, and so on. The cell component is related to extracellular exosome, extracellular space, extracellular region, cytosol, extracellular matrix, blood microparticle, focal adhesion, muscle myosin complex, membrane, sarcomere, melanosome, stress fiber, platelet alpha granule lumen, actin filament, cell-cell adherens junction, myosin filament, Z disc, basement membrane, and so on. The molecular function is related to structural constituent of muscle, glycoprotein binding, identical protein binding, antioxidant activity, calcium ion binding, serine-type endopeptidase activity, actin binding, cadherin binding involved in cell-cell adhesion, MHC class II protein complex binding, protein binding, poly(A) RNA binding, unfolded protein binding, virion binding, endopeptidase inhibitor activity, actin filament binding, cytoskeletal protein binding, serine-type endopeptidase inhibitor activity, and so on. The signaling pathway is mainly related to metabolic pathways, biosynthesis of amino acids, glycolysis/gluconeogenesis, arginine biosynthesis, carbon metabolism, and so on (Figure 6). Their details were shown in Table S2.

### 3.3. Chemoinformatics Analysis Results

#### 3.3.1. Edaravone-CI PPI Network

**Figure 7:**
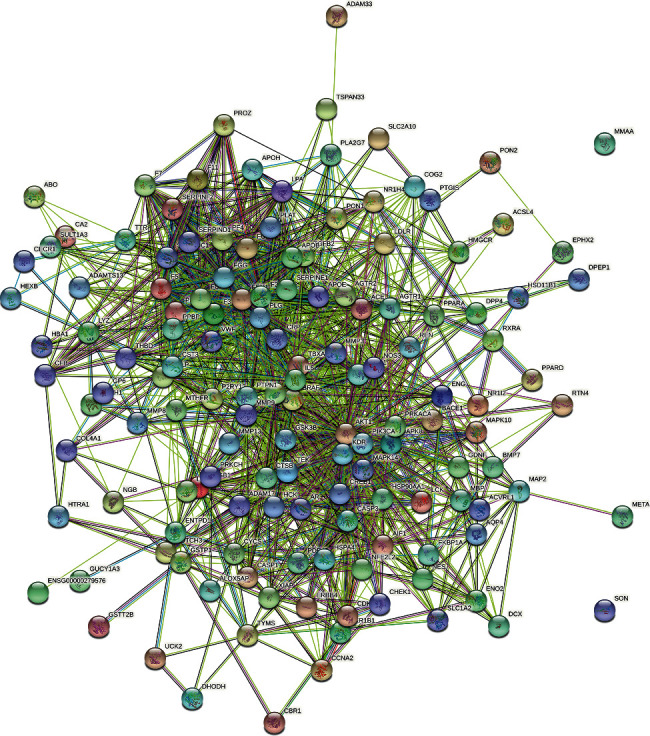
Edaravone-CI PPI network.

Edaravone target and CI gene were introduced into String to construct edaravone-CI PPI network. This network is composed of 141 nodes and 1324 edges. The average node degree is 18.8, and the average local clustering coefficient is 0.526 (Figure 7).

#### 3.3.2. Enrichment Analysis Results

**Figure 8:**
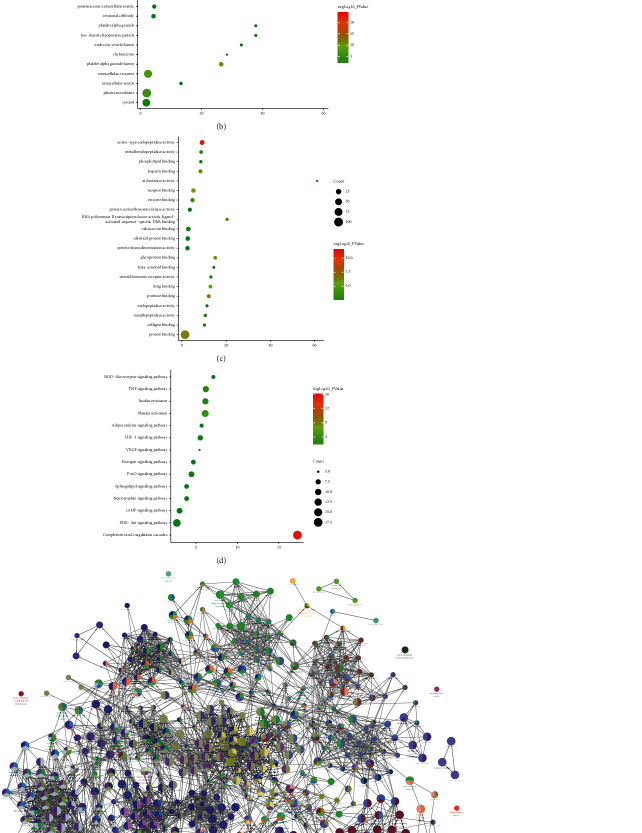
The results of enrichment analysis (a: biological processes; b: cell components; c: molecular function; d: signaling pathways; *X*-axis stands for fold enrichment. e and f: Cluego analysis).

The Edaravone target and CI gene were input into David for enrichment analysis and returns 14 CI-related signaling pathways, 63 biological processes, 21 cell components, and 21 molecular function. Their *P* value, fold enrichment and count of each signaling pathway, biological process, cell component, and molecular function are shown in Figure 8. The biological process is related to blood coagulation, platelet activation, proteolysis, platelet degranulation, fibrinolysis, response to hypoxia, and so on. The cell component is related to extracellular space, extracellular region, platelet alpha granule lumen, cell surface, extracellular matrix, extracellular exosome, and so on. The molecular function is related to serine-type endopeptidase activity, protease binding, protein binding, heparin binding, glycoprotein binding, receptor binding, and so on. The signaling pathway is related to complement and coagulation cascades, platelet activation, TNF signaling pathway, insulin resistance, HIF-1 signaling pathway, FoxO signaling pathway, and so on (Figure 8). Their details were shown in Table S3.

### 3.4. Expression of Differentially Expressed Proteins in Brain Tissue

The expression of DEPs in each organ in each comparison group was analyzed, and the obtained DEPs were analyzed by Expression Atlas. After the tissue specificity is set to “brain,” the protein with the number 10 in descending order or the protein with number ≧10 is matched. The brain tissue specificity of these proteins is ranked according to their expression strength in brain tissue and other tissues, and the detailed information is listed in Tables 4 and 5.

Different proteins with upregulated expression and high brain specificity in the CI/CE comparison group are cathepsin D, LOC683667 protein, galectin-1, C-reactive protein, and 40S ribosomal protein S6.

Different proteins with downregulated expression and high brain specificity in the CI/CN comparison group are cystatin-C, serotransferrin, peptidyl-prolyl cis-trans isomerase, beta-2-microglobulin, carbonic anhydrase2, angiotensinogen, peroxiredoxin-6, glutathione peroxidase, 4-trimethylaminobutyraldehyde dehydrogenase, and catalase.

### 3.5. Oxidative Stress Indicators in Brain Tissue

**Figure 9:**
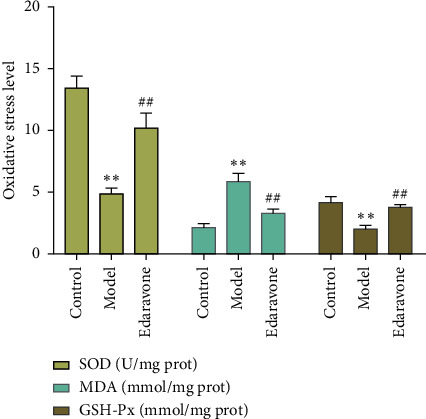
Oxidative stress indicators in brain tissue (^∗∗^compared with sham operation group, *P* < 0.05; ##compared with model group, *P* < 0.05).

Compared with the sham operation group, the MDA content of the model group was significantly increased, while the SOD and GSH content were significantly decreased (*P* < 0.05), indicating that the model was successful. Compared with the model group, the MDA content of the edaravone administration group significantly decreased, and the SOD and GSH content significantly increased (*P* < 0.05), indicating that edaravone can improve the brain SOD, MDA, and GSH content of rats (Figure 9).

## 4. Discussion

CI is a cerebrovascular disease characterized by brain blood supply disorder, local avascular necrosis, or softening of brain tissue caused by ischemia and hypoxia. Among them, neuronal programmed death (apoptosis, autophagy, iron death, scorch death, etc.) and necrosis exist simultaneously. The main pathological mechanisms include energy exhaustion, excitotoxicity, oxidative stress, endoplasmic reticulum dysfunction, and a large amount of immune cell infiltration and aggregation. Various cytokines are involved in a series of “cascade-like” cascade events, such as neuroinflammation in the infarct area, which ultimately leads to the death of nerve cells. The damage caused by free radicals induced by oxidative stress is considered to be an important pathological basis for brain neuron damage in the onset of stroke. The chain reaction of free radicals causes excessive oxidation of cell membrane lipids and nuclear proteins and damages the structure and function of mitochondria, resulting in insufficient cell energy production, leading to cell death or apoptosis. Therefore, antioxidative stress response and scavenging excess free radicals may be important strategies for early CI to protect neurons and reduce damage.

Edaravone, as a free radical scavenger, has the effects of antioxidative stress and inhibiting lipid peroxidation. Studies have confirmed that edaravone can protect body tissues by inhibiting the activity of cyclooxygenase (COX, including COX-1, and COX-2). COX-2 is mainly expressed in hippocampal tissue and cortical neurons and can mediate neuronal damage. COX-2 is a key enzyme and rate-limiting enzyme produced by prostaglandin E2 (PGE2) metabolism, and it can also promote the production of nitric oxide (NO), while PGE2 and NO can participate in various pathological processes, such as inflammation, oxidative stress, and apoptosis. Current clinical and basic research shows that edaravone, which has an antioxidant effect, may become a new type of drug for the treatment of CI. This study established the CI rat model and used serum proteomics to determine the serum markers for edaravone to interfere with CI. 153 differentially expressed proteins include Rps6, Spp2, Lgals1, Pros1, Aldh9a1, Fcn2, Serpinf1, Asl, Agt, Cat, Ckm, Pfkfb1, Fbp1, Aldh1l1, Irak2, Gk, Mat1a, Pah, Tf, Adh1, Bhmt, Cps1, Prss1, LOC103691744, Tpm1, and Myh1. Recent studies have shown that ribosomal protein S6 (rpS6), extracellular signal-regulated mitogen-activated protein (MAP) kinase p44/42 (p44/42MAPK), and ribosomal protein S6 kinase (S6K) are significantly reduced in the brains of hibernating squirrels and rats. Therefore, we believe that the downregulation of rpS6 signal transduction may be an important reason for the increased cell tolerance to cerebral ischemia observed during hibernation numbness and after ischemic preconditioning, by inhibiting protein synthesis and/or energy expenditure [24]. Qu et al. showed that galectin-1 (Gal-1) can regulate the proliferation of a variety of cells and play an important role after nervous system injury. Galectin-1 can reduce astrocyte damage and improve the recovery of rats with focal cerebral ischemia [25]. Ishibashi S. et al. also found that galectin-1 regulates neurogenesis in the subventricular zone and promotes functional recovery after stroke [26]. Agt is the gene encoding angiotensinogen, and current studies have shown that its polymorphism is closely related to stroke, especially cerebral small vessel disease [27–29]. Current research shows that Catalase (CAT) has important physiological functions in oxidative stress. CAT can quickly remove the toxic substances produced by cell metabolism, so as to protect sulfhydrylase and membrane protein and detoxify together with glutathione peroxidase (GSHPx). It plays an important protective role against cerebral ischemia in CI patients [30, 31]. Aldh1l1 is mainly related to the proliferation of astrocytes after CI [32]. Interleukin-1 receptor-associated kinases (IRAKs) are mainly related to inflammation and cell apoptosis after cerebral ischemia and reperfusion [33].

The enrichment analysis of these 153 differentially expressed proteins involved a total of 12 signal pathways, 84 biological processes, 41 cell components, and 29 molecular functions. The biological process is mainly in platelet activation and aggregation (GO:0002576∼platelet degranulation, GO:0070527∼platelet aggregation), oxidative stress (GO:0098869∼cellular oxidant detoxification; GO:0006986∼response to unfolded protein; GO:0042542∼response to hydrogen peroxide; GO:0000302∼response to reactive oxygen species; GO:0090201∼negative regulation of release of cytochrome c from mitochondria; GO:0042744∼hydrogen peroxide catabolic process; GO:0045454∼cell redox homeostasis), intercellular adhesion (GO:0098609∼cell-cell adhesion), glycolysis and gluconeogenesis (GO:0061621∼canonical glycolysis; GO:0006094∼gluconeogenesis), iron metabolism (GO:0033572∼transferrin transport; GO:0071281∼cellular response to iron ion), hypoxia (GO:0001666∼response to hypoxia), inflammatory chemokines and their mediated signal transduction (GO:0050729∼positive regulation of inflammatory response; GO:0051092∼positive regulation of NF-kappaB transcription factor activity; GO:0032602∼chemokine production) reaction, coagulation (GO:0030194∼positive regulation of blood coagulation; GO:0007597∼blood coagulation, intrinsic pathway), apoptosis (GO:0043066∼negative regulation of apoptotic process; GO:1902175∼ regulation of oxidative stress-induced intrinsic apoptotic signaling pathway), fibrinolysis system (GO:0042730∼fibrinolysis; GO:0051919∼positive regulation of fibrinolysis); angiogenesis (GO:0016525∼negative regulation of angiogenesis) endoplasmic reticulum stress (GO:0034976∼response to endoplasmic reticulum stress), and energy metabolism (GO:0046034∼ATP metabolic process; GO:0045040∼protein import into mitochondrial outer membrane). These biological modules play an important role in the acute pathological process of cerebral infarction.

The molecular functions also focused on oxidative stress (GO:0016209∼antioxidant activity; GO:0004029∼aldehyde dehydrogenase (NAD) activity; GO:0016620∼oxidoreductase activity, acting on the aldehyde or oxo group of donors, NAD or NADP as acceptor), calcium ion binding (GO:0005509∼calcium ion binding; GO:0098641∼cadherin binding involved in cell-cell adhesion), energy metabolism (GO:0005524∼ATP binding), inflammation (GO:0050544∼arachidonic acid binding), and other molecular functions. The cellular components focus on exosomes (GO:0070062∼extracellular exosome), extracellular space (GO:0005615∼extracellular space.; GO:0031012∼extracellular matrix), blood particles (GO:0072562∼blood microparticle), intercellular adhesion (GO:0005925∼focal adhesion; 5913∼cell-cell adherens junction), platelet (GO:0031093∼platelet alpha granule lumen; GO:0031089∼platelet dense granule lumen), endoplasmic reticulum cavity (GO:0005788), and myelin sheath (GO:0043209).

Oxidative stress is considered to be an important factor leading to cell damage in acute stroke events. During the period of cerebral ischemia and perfusion, especially the reperfusion period, a large number of free radicals are produced, which attack the lipids and proteins of cell membranes [34, 35]. In recent years, studies have found that in addition to directly attacking cell membranes and causing cell oxidative damage, free radicals can also participate in the process of neuronal damage by initiating apoptosis pathways. The free radicals produced after cerebral ischemia can act on mitochondria and change the permeability of mitochondrial pores. The depolarization of the mitochondrial membrane leads to the release of cytochrome c from mitochondria into the cytoplasm, which activates the caspase family to cause DNA damage and lead to apoptosis [36, 37]. In addition, the reactive oxygen species produced by oxidative stress can also regulate the activity of nuclear transcription factor NF-kB and affect the downstream genes of NF-kB, including iNOs, COX-2, intercellular adhesion molecules, and cytokines [38–41]. They mediate the destruction of blood-brain barrier function and inflammation and play an important role in the signal transduction of apoptosis [42–44]. Therefore, after cerebral infarction and its reperfusion, the overproduction and burst of free radicals play a key role in the programmed death of neurons. Edaravone is an effective antioxidant, which can inhibit the oxidation of NO and increase the production of NO by increasing the expression of eNOS and inhibit the formation of peroxygen during reperfusion to improve or protect vascular blood flow [45]. According to recent reports, in MCAO model mice, edaravone administered within 6 hours of reperfusion can reduce infarct volume and improve neurological deficits. In the early stage of ischemic events, edaravone can inhibit the accumulation of 4-hydroxy-2-nonenal (HNE) modified protein and 8-hydroxydeoxyguanosine (8-OHdG) in the ischemic penumbra. In the later period, it reduces the activation of microglia, the expression of iNOS, and the formation of astrocytes peroxygen [46]. Jiao et al. found that edaravone can reduce the delayed neuronal death of the hippocampus after middle cerebral artery ischemia reperfusion, and its mechanism may be related to scavenging free radicals, resisting inflammation, and inhibiting the activation of astrocytes [47]. In terms of cerebral hemorrhage, edaravone may inhibit the activation of M1 type microglia and the accumulation of proinflammatory factors through the IRE1*α*/TRAF2 signaling pathway after ER stress, reduce the edema and apoptosis of nerve cells after cerebral hemorrhage in mice, reduce the damage of white matter fiber bundles, and then protect nerve function [48]. In terms of the proliferation and differentiation of neural stem cells, edaravone can promote the proliferation of endogenous neural stem cells and astrocytes in the subventricular area of the ischemic injury side and the cerebral cortex around ischemia and promote the differentiation of endogenous neural stem cells into neurons [49]. Further studies have shown that edaravone promotes neuronal proliferation in acute stroke rats through the Wnt/*β*-catenin signaling pathway and improves the neurological and cognitive functions of acute stroke rats [50]. In terms of iron metabolism, the effect of edaravone in repairing spinal cord injury in rats is achieved by regulating the xCT/GPX4/ACSL4/5-LOX iron death pathway and rebalancing the homeostasis of lipid oxidation. Further studies have shown that edaravone reduces 5-LOX metabolites to inhibit neuroinflammation, promote neuronal survival after spinal cord injury, and prevent iron death in neuronal cell lines [51]. Compared with previous studies, this study revealed the serum protein profile of edaravone in the treatment of cerebral infarction rats through serum TMT proteomics and discovered the relevant mechanism of edaravone regulating iron metabolism in cerebral infarction, which provides new ideas for the study of edaravone intervention in cerebral infarction and also provides reference information for future research on the mechanism of edaravone intervention in iron metabolism-related diseases.

## 5. Conclusion

The therapeutic mechanism of edaravone in the treatment of CI may involve platelet activation and aggregation, oxidative stress, intercellular adhesion, glycolysis and gluconeogenesis, iron metabolism, hypoxia, and so on. This study provides new reference for the clinical application of edaravone for CI.

## Figures and Tables

**Table 1:** Neurological score.

Group	Score (*X* ± *S*)	*P* value
CN	0	*—*
CI	2.813 ± 0.403	—
CE	2.067 ± 0.799	0.0012

**Table 2:** Differential expressed protein of CI/CN group.

Protein description	Gene name	CI/CN ratio	Regulated type
Endoplasmin	Hsp90b1	1.395	Up
4-Trimethylaminobutyraldehyde dehydrogenase	Aldh9a1	1.67	Up
Betaine-homocysteine S-methyltransferase 1	Bhmt	4.005	Up
Creatine kinase M-type	Ckm	2.532	Up
Calpastatin	Cast	1.368	Up
Pyruvate kinase	Pkm	1.667	Up
Protein IGHM	IGHM	0.707	Down
COP9 signalosome complex subunit 1	Gps1	1.65	Up
Transgelin	Tagln	1.605	Up
Insulin-like growth factor I	Igf1	0.632	Down
Protein P4ha2	P4ha2	1.457	Up
Polyadenylate-binding protein 1	Pabpc1	1.544	Up
“6-Phosphofructo-2-kinase/fructose-2,6-bisphosphatase 1”	Pfkfb1	1.723	Up
Protein F5	F5	0.692	Down
Protein Myh1	Myh1	2.155	Up
Protein Ugp2	Ugp2	1.635	Up
Protein LOC103691744	LOC103691744	4.174	Up
Protein Myom1	Myom1	1.745	Up
Protein RGD1564614	RGD1564614	0.711	Down
Protein LOC299282	Serpina3n	1.632	Up
Protein S100-A9	S100a9	1.873	Up
Haptoglobin	Hp	1.9	Up
Filamin-C	Flnc	1.991	Up
“Ubiquilin 1, isoform CRA_a”	Ubqln1	1.658	Up
Myl9 protein	Myl9	1.792	Up
Filamin alpha	Flna	1.81	Up
Matrix metalloproteinase 19	Mmp19	0.724	Down
Glycerol kinase	Gk	2.08	Up
Protein papln	Papln	0.734	Down
Calponin	Cnn2	0.714	Down
Protein aldh8a1	Aldh8a1	1.784	Up
Neutrophilic granule protein (predicted)	Ngp	1.729	Up
Protein col14a1	Col14a1	2.295	Up
Protein Atp6v1a	Atp6v1a	1.341	Up
Protein LOC100362751	LOC498555	2.379	Up
Leukocyte cell-derived chemotaxin 2 (predicted)	Lect2	0.739	Down
Protein Rsu1	Rsu1	0.675	Down
“Fructose-1,6-bisphosphatase 1”	Fbp1	1.709	Up
Heat shock 70 kDa protein 4	Hspa4	1.526	Up
Protein Myh1	Myh1	1.987	Up
S-adenosylmethionine synthase	Mat1a	1.787	Up
Protein Igkv8-27	Igkv8-27	0.536	Down
Protein Lama2	Lama2	1.429	Up
Protein Rrbp1	Rrbp1	2.254	Up
Protein Lamc1	Lamc1	2.232	Up
“Nitrilase 1, isoform CRA_a”	Nit1	1.789	Up
Protein Myh1	Myh2	2.763	Up
Myomesin 2	Myom2	2.09	Up
“ATPase, H+ transporting, V1 subunit E isoform 1, isoform CRA_a”	Atp6v1e1	1.351	Up
Lamin-B1	Lmnb1	2.227	Up
“Prolactin induced protein, isoform CRA_d”	Pip	2.152	Up
Myosin-7	Myh7	2.873	Up
“Lamin A, isoform CRA_b”	Lmna	2.146	Up
Integrin alpha M	Itgam	1.393	Up
“RCG39455, isoform CRA_a”	Tsku	0.602	Down
Acylamino-acid-releasing enzyme	Apeh	0.65	Down
ATP-citrate synthase	Acly	1.686	Up
Protein Sec24d	Sec24d	1.551	Up
“Protein S (alpha), isoform CRA_b”	Pros1	0.679	Down
40S ribosomal protein S6	Rps6	0.535	Down
Ras-related protein Rab-11B	Rab11b	1.566	Up
Bifunctional purine biosynthesis protein PURH	Atic	1.777	Up
Ribonuclease 4	Rnase4	0.762	Down
Anionic trypsin 1	Prss1	4.631	Up
Angiotensinogen	Agt	1.777	Up
“Myosin light chain 1/3, skeletal muscle isoform”	Myl1	2.052	Up
“Myosin regulatory light chain 2, skeletal muscle isoform”	Mylpf	2.39	Up
Tropomyosin alpha-1 chain	Tpm1	2.41	Up
Catalase	Cat	1.669	Up
Protein disulfide-isomerase	P4hb	1.408	Up
Fructose-bisphosphate aldolase A	Aldoa	1.927	Up
Elongation factor 2	Eef2	1.652	Up
Delta-aminolevulinic acid dehydratase	Alad	1.858	Up
Alcohol dehydrogenase 1	Adh1	2.348	Up
“Carbamoyl-phosphate synthase [ammonia], mitochondrial”	Cps1	4.184	Up
Tropomyosin alpha-4 chain	Tpm4	0.647	Down
“Glycogen phosphorylase, liver form”	Pygl	1.704	Up
Adenosylhomocysteinase	Ahcy	1.729	Up
Galectin-1	Lgals1	0.599	Down
Serotransferrin	Tf	1.471	Up
cAMP-dependent protein kinase type II-beta regulatory subunit	Prkar2b	0.506	Down
Insulin-like growth factor-binding protein 2	Igfbp2	0.661	Down
“Phospholipase A2, membrane associated”	Pla2g2a	0.562	Down
Phosphoglycerate kinase 1	Pgk1	1.581	Up
60S acidic ribosomal protein P0	Rplp0	2.136	Up
Insulin-like growth factor-binding protein 1	Igfbp1	0.56	Down
Cytosolic 10-formyltetrahydrofolate dehydrogenase	Aldh1l1	1.968	Up
Protein S100-A8	S100a8	1.535	Up
Ficolin-2	Fcn2	1.54	Up
Tropomyosin beta chain	Tpm2	3.257	Up
14-3-3 protein epsilon	Ywhae	1.607	Up
Heat shock cognate 71 kDa protein	Hspa8	2.161	Up
“60 kDa heat shock protein, mitochondrial”	Hspd1	1.749	Up
“Actin, alpha cardiac muscle 1”	Actc1	1.579	Up
“Actin, alpha skeletal muscle”	Acta1	3.221	Up
Heat shock protein HSP 90-alpha	Hsp90aa1	1.606	Up
Pleckstrin	Plek	0.638	Down
Interleukin-1 receptor-associated kinase-like 2	Irak2	0.447	Down
Argininosuccinate lyase	Asl	1.607	Up
Aspartyl aminopeptidase	Dnpep	1.473	Up
Tubulin alpha-4A chain	Tuba4a	0.683	Down
Ras-related protein Rap-1b	Rap1b	0.691	Down
Secreted phosphoprotein 24	Spp2	0.521	Down
Nucleobindin-1	Nucb1	1.625	Up
Receptor-type tyrosine-protein phosphatase F	Ptprf	1.443	Up
COP9 signalosome complex subunit 4	Cops4	1.441	Up
COP9 signalosome complex subunit 3	Cops3	1.787	Up
Group specific component	Gc	2.016	Up
Carboxypeptidase	Ctsa	1.415	Up
Phenylalanine hydroxylase	Pah	2.261	Up
Nucleotide exchange factor SIL1	Sil1	1.666	Up
Desmin	Des	2.285	Up
Alpha-2 antiplasmin	Serpinf1	1.488	Up
Aflatoxin B1 aldehyde reductase member 2	Akr7a2	1.647	Up
“Tropomyosin 1, alpha, isoform CRA_a”	Tpm1	3.376	Up
Hepcidin	Hamp	1.643	Up
Guanine deaminase	Gda	1.566	Up
Peroxiredoxin-4	Prdx4	1.601	Up

**Table 3:** Differential expressed protein of CE/CI group.

Protein description	Gene name	CE/CI ratio	Regulated type
4-Trimethylaminobutyraldehyde dehydrogenase	Aldh9a1	0.739	Down
Betaine-homocysteine S-methyltransferase 1	Bhmt	0.42	Down
Creatine kinase M-type	Ckm	0.637	Down
Galectin	Lgals3	1.564	Up
Thyroglobulin	Tg	2.708	Up
“6-Phosphofructo-2-kinase/fructose-2,6-bisphosphatase 1”	Pfkfb1	0.62	Down
Complement factor I	Cfi	0.574	Down
Protein TNC	TNC	1.606	Up
Peptidyl-prolyl cis-trans isomerase	Ppia	0.729	Down
Protein F11	F11	1.337	Up
Protein LOC103691744	LOC103691744	0.204	Down
Protein RGD1310507	RGD1310507	0.697	Down
Histidine-rich glycoprotein	Hrg	5.24	Up
Coagulation factor XII	F12	0.669	Down
LOC683667 protein	Sri	1.321	Up
Glycerol kinase	Gk	0.537	Down
Protein Fat4	Fat4	1.45	Up
Carboxylic ester hydrolase	Ces1c	0.421	Down
RCG21066	rCG_21066	0.686	Down
Protein IGHM	IGHM	1.411	Up
“Sushi, Von Willebrand factor type A, EGF and pentraxin domain-containing protein 1”	Svep1	1.454	Up
“Procollagen, type XVIII, alpha 1, isoform CRA_a”	Col18a1	1.499	Up
Serine protease inhibitor A3M	Serpina3m	0.616	Down
“Fructose-1,6-bisphosphatase 1”	Fbp1	0.602	Down
Serine protease hepsin	Hpn	1.413	Up
S-adenosylmethionine synthase	Mat1a	0.521	Down
“Troponin I, fast skeletal muscle”	Tnni2	0.722	Down
“Fibulin 2, isoform CRA_a”	Fbln2	1.354	Up
Apolipoprotein C-II (predicted)	Apoc2	2.066	Up
Heat shock 27 kDa protein 1	Hspb1	1.671	Up
“Protein S (alpha), isoform CRA_b”	Pros1	1.448	Up
Protein LOC691828	LOC691828	0.718	Down
Glutathione peroxidase	Gpx1	0.399	Down
40S ribosomal protein S6	Rps6	2.075	Up
Peroxiredoxin-6	Prdx6	0.582	Down
Lysozyme C-1	Lyz1	0.675	Down
Anionic trypsin 1	Prss1	0.272	Down
Angiotensinogen	Agt	0.687	Down
Serum albumin	Alb	0.67	Down
Catalase	Cat	0.658	Down
Alcohol dehydrogenase 1	Adh1	0.442	Down
Beta-2-microglobulin	B2m	0.656	Down
“Carbamoyl-phosphate synthase [ammonia], mitochondrial”	Cps1	0.297	Down
Galectin-1	Lgals1	1.658	Up
Cysteine-rich secretory protein 1	Crisp1	0.439	Down
Serotransferrin	Tf	0.471	Down
Cystatin-C	Cst3	0.625	Down
Carbonic anhydrase 2	Ca2	0.533	Down
Cytosolic 10-formyltetrahydrofolate dehydrogenase	Aldh1l1	0.599	Down
C-reactive protein	Crp	1.454	Up
Cadherin-17	Cdh17	1.512	Up
Ficolin-2	Fcn2	0.724	Down
Interleukin-1 receptor-associated kinase-like 2	Irak2	0.556	Down
Argininosuccinate lyase	Asl	0.707	Down
Apolipoprotein H	Apoh	0.644	Down
Protein Serpina4	Serpina4	0.727	Down
Protein Hbb-b1	LOC103694855	0.507	Down
Secreted phosphoprotein 24	Spp2	1.849	Up
BPI fold-containing family a member 2	Bpifa2	0.46	Down
Phenylalanine hydroxylase	Pah	0.491	Down
Cathepsin D	Ctsd	1.466	Up
Alpha-2 antiplasmin	Serpinf1	0.713	Down

**Table 4:** DEPs with higher expression in brain tissue in the CI/CE operation comparison group.

Gene name	Adrenal gland	Brain	Gastrocnemius	Heart	Kidney	Liver	Lung	Spleen	Testis	Thymus
Ctsd	297	167	154	126	258	125	651	195	35	182
Sri	28	45	11	16	68	13	100	146	144	85
Lgals1	311	37	254	156	42	6	274	79	65	161
Crp	20	27	9	8	15	1447	27	19	21	24
Rps6	30	24	18	18	22	24	50	145	29	98

**Table 5:** DEPs with higher expression in brain tissue in the CI/CN comparison group.

Gene name	Adrenal gland	Brain	Gastrocnemius	Heart	Kidney	Liver	Lung	Spleen	Testis	Thymus
Cst3	279	1558	273	279	240	112	722	495	381	638
Tf	42	447	41	24	43	18878	109	126	127	41
Ppia	183	308	37	54	217	181	207	269	236	413
B2m	685	186	166	558	507	1168	1661	1926	156	1003
Car2	27	183	12	11	250	6	101	293	40	12
Agt	47	96	2	2	13	372	31	6	0.7	51
Prdx6	108	68	28	59	103	79	292	41	46	54
Gpx1	347	52	160	330	635	1326	375	669	27	189
Aldh9a1	62	21	28	30	151	258	46	33	29	41
Cat	146	21	48	46	379	1270	113	81	8	56

## Data Availability

The data that support the findings of this study are openly available in supplementary materials.

## Supplementary Materials

Supplementary Materials Table S1: edaravone potential targets and CI genes. Table S2: enrichment analysis results of proteomics. Table S3: enrichment analysis results of edaravone-CI PPI network
