# Supplementary material for: Exploring the Mechanism of Edaravone for Oxidative Stress in Rats with Cerebral Infarction Based on Quantitative Proteomics Technology
Source: Evid Based Complement Alternat Med. 2022 Jan 4;2022:8653697. doi: 10.1155/2022/8653697 (PMC8752268; doi:10.1155/2022/8653697)
Supplement: Supplementary Materials — Table S1: edaravone potential targets and CI genes. Table S2: enrichment analysis results of proteomics. Table S3: enrichment analysis results of edaravone-CI PPI network [file 8653697.f1.zip › 8653697.f1/Table S1.pdf]

Table S1 Edaravone Potential Targets and CI genes

Group	Target or Genes
Edaravone	MAPK10
	MAPK8
	MMP3
	CFB
	F2
	PPARD
	CTSB
	HSD11B1
	CBR1
	CHEK1
	CA2
	METAP2
	MMP8
	HSP90AA1
	GSK3B
	MAPK14
	DHODH
	DPP4
	ADAM17
	CDK2
	MMP13
	RXRA
	KDR
	PTPN1
	ERBB4
	UCK2
	ACE
	GSTT2B
	NR1I2
	XIAP
	CCNA2
	FKBP1A
	PDE4D
	AR
	DPEP1
	F11
	TTR
	LYZ
	BRAF
	TYMS
	HCK
	NR1H4
	HEXB
	HMGCR
	TGFB2
	REN

	PRKACA
	CASP1
	TEK
	MMP1
	PPARA
	LCK
	ADAM33
	GSTP1
	BACE1
	ABO
	FGG
	CASP3
	AKR1B1
CI	F5
	F2
	NOS3
	NOTCH3
	ALOX5AP
	MTHFR
	PRKCH
	FBN1
	LDLR
	CRP
	ACE
	COL4A1
	SERPINE1
	SELP
	PLAT
	APOE
	PDE4D
	PIK3CA
	ACSL4
	MT-TL1
	PON1
	VWF
	HTRA1
	FGA
	APOH
	LPA
	AGTR1
	APOB
	SERPINC1
	F7
	ADAMTS13
	PLA2G7
	EPHX2
	GUCY1A1

ENO2
CDKN2B-AS1
TLR4
THBD
PON2
F3
PROZ
MALAT1
COG2
MROS
P2RY12
REN
GP6
SULT1A3
TBXA2R
HMGB1
ACVRL1
AQP4
CST3
NGB
CASP3
ADA2
MBP
ENG
LOX
PLG
PTGIS
SLC1A2
MIAT
HMGCR
PF4
MMAA
FGB
HBA1
HSPA4
NES
SERPINF2
CREB1
PPBP
CYCS
SERPIND1
RTN4
AGTR2
MAP2
SLC2A10
IL6
AKT1
GDNF

NFE2L2
ENTPD1
ASAH1
BMP7
DCX
AIF1
TSPAN33
SON
PROC
MMP9
