# Supplementary material for: Exploring the Mechanism of Edaravone for Oxidative Stress in Rats with Cerebral Infarction Based on Quantitative Proteomics Technology
Source: Evid Based Complement Alternat Med. 2022 Jan 4;2022:8653697. doi: 10.1155/2022/8653697 (PMC8752268; doi:10.1155/2022/8653697)
Supplement: Supplementary Materials — Table S1: edaravone potential targets and CI genes. Table S2: enrichment analysis results of proteomics. Table S3: enrichment analysis results of edaravone-CI PPI network [file 8653697.f1.zip › 8653697.f1/Table S2.pdf]

Table S2 Enrichment ana

Category	Term
	GO:0030049
	GO:0002576
	GO:0006936
	GO:0098869
	GO:0006508
	GO:0010951
	GO:0042493
	GO:0010043
	GO:0045471
	GO:0042026
	GO:0001895
	GO:0006986
	GO:0098609
	GO:0060048
	GO:0042542
	GO:0003009
	GO:0061621
	GO:0050832
	GO:0010269
	GO:0006730
	GO:0010388
	GO:0007568
	GO:0006000
	GO:0006641
	GO:0033572
	GO:0001666
	GO:0050729
	GO:0000096
	GO:0000302
	GO:0070527
	GO:0006195
	GO:0051131
	GO:0014823
	GO:0030198
	GO:0006094
	GO:0030194
	GO:0006937
	GO:0043254
	GO:0007517
	GO:0090201
	GO:0007597
	GO:0042744
	GO:0043066
	GO:0042730
	GO:0051289
	GO:0016525

	GO:0000715
	GO:0044267
	GO:0043627
	GO:0051384
	GO:0070488
	GO:0032868
	GO:0046686
	GO:0010628
	GO:0007015
	GO:0034976
	GO:0051092
	GO:1902175
	GO:0061684
	GO:0032602
	GO:0045454
	GO:0008016
	GO:0042060
	GO:0046034
	GO:0042742
	GO:0006096
	GO:0030307
	GO:0002793
	GO:0051919
	GO:0032119
	GO:0051450
	GO:0050830
	GO:0006928
	GO:2001237
	GO:0032355
	GO:0034392
	GO:0045040
	GO:0090131
	GO:0010756
	GO:0030240
	GO:0006526
	GO:0006953
	GO:0071281
**BP**	GO:2001200
	GO:0070062
	GO:0005615
	GO:0005576
	GO:0005829
	GO:0031012
	GO:0072562
	GO:0005925
	GO:0005859
	GO:0016020

	GO:0030017
	GO:0042470
	GO:0001725
	GO:0031093
	GO:0005884
	GO:0005913
	GO:0032982
	GO:0030018
	GO:0005604
	GO:0005862
	GO:0005788
	GO:0036464
	GO:0043209
	GO:0043202
	GO:0031089
	GO:0071682
	GO:0048471
	GO:0031430
	GO:0098575
	GO:0030016
	GO:0030529
	GO:0045121
	GO:0031941
	GO:0016324
	GO:0016235
	GO:0008180
	GO:0005638
	GO:0016469
	GO:0005623
	GO:0005911
	GO:0014704
**CC**	GO:0005783
	GO:0008307
	GO:0001948
	GO:0042802
	GO:0016209
	GO:0005509
	GO:0004252
	GO:0003779
	GO:0098641
	GO:0023026
	GO:0005515
	GO:0044822
	GO:0051082
	GO:0046790
	GO:0004866
	GO:0051015

	GO:0008092
	GO:0004867
	GO:0004029
	GO:0016620
	GO:0005524
	GO:0060230
	GO:0004869
	GO:0019145
	GO:0004656
	GO:0047105
	GO:0035662
	GO:0050544
	GO:0005179
**MF**	GO:0051920
	hsa01230
	hsa05410
	hsa05414
	hsa01130
	hsa04510
	hsa04141
	hsa00010
	hsa04610
	hsa01200
	hsa04260
	hsa01100
**PY**	hsa04966

lysis Results of Proteomics

Description	Count
muscle filament sliding	10
platelet degranulation	13
muscle contraction	13
cellular oxidant detoxification	7
proteolysis	15
negative regulation of endopeptidase activity	8
response to drug	11
response to zinc ion	5
response to ethanol	7
protein refolding	4
retina homeostasis	5
response to unfolded protein	5
cell-cell adhesion	10
cardiac muscle contraction	5
response to hydrogen peroxide	5
skeletal muscle contraction	4
canonical glycolysis	4
defense response to fungus	4
response to selenium ion	3
one-carbon metabolic process	4
cullin deneddylation	3
aging	7
fructose metabolic process	3
triglyceride metabolic process	4
transferrin transport	4
response to hypoxia	7
positive regulation of inflammatory response	5
sulfur amino acid metabolic process	3
response to reactive oxygen species	4
platelet aggregation	4
purine nucleotide catabolic process	3
chaperone-mediated protein complex assembly	3
response to activity	4
extracellular matrix organization	7
gluconeogenesis	4
positive regulation of blood coagulation	3
regulation of muscle contraction	3
regulation of protein complex assembly	3
muscle organ development	5
negative regulation of release of cytochrome c from mitochondria	3
blood coagulation, intrinsic pathway	3
hydrogen peroxide catabolic process	3
negative regulation of apoptotic process	10
fibrinolysis	3
protein homotetramerization	4
negative regulation of angiogenesis	4

nucleotide-excision repair, DNA damage recognition	3
cellular protein metabolic process	5
response to estrogen	4
response to glucocorticoid	4
neutrophil aggregation	2
response to insulin	4
response to cadmium ion	3
positive regulation of gene expression	7
actin filament organization	4
response to endoplasmic reticulum stress	4
positive regulation of NF-kappaB transcription factor activity	5
regulation of oxidative stress-induced intrinsic apoptotic signaling pathway	2
chaperone-mediated autophagy	2
chemokine production	2
cell redox homeostasis	4
regulation of heart contraction	3
wound healing	4
ATP metabolic process	3
defense response to bacterium	5
glycolytic process	3
positive regulation of cell growth	4
positive regulation of peptide secretion	2
positive regulation of fibrinolysis	2
sequestering of zinc ion	2
myoblast proliferation	2
defense response to Gram-positive bacterium	4
movement of cell or subcellular component	4
negative regulation of extrinsic apoptotic signaling pathway	3
response to estradiol	4
negative regulation of smooth muscle cell apoptotic process	2
protein import into mitochondrial outer membrane	2
mesenchyme migration	2
positive regulation of plasminogen activation	2
skeletal muscle thin filament assembly	2
arginine biosynthetic process	2
acute-phase response	3
cellular response to iron ion	2
positive regulation of dendritic cell differentiation	2

extracellular exosome	95
extracellular space	52
extracellular region	50
cytosol	69
extracellular matrix	23
blood microparticle	14
focal adhesion	17
muscle myosin complex	6
membrane	38

sarcomere	6
melanosome	8
stress fiber	6
platelet alpha granule lumen	6
actin filament	6
cell-cell adherens junction	11
myosin filament	4
Z disc	7
basement membrane	6
muscle thin filament tropomyosin	3
endoplasmic reticulum lumen	8
cytoplasmic ribonucleoprotein granule	4
myelin sheath	7
lysosomal lumen	5
platelet dense granule lumen	3
endocytic vesicle lumen	3
perinuclear region of cytoplasm	12
M band	3
lumenal side of lysosomal membrane	2
myofibril	3
intracellular ribonucleoprotein complex	5
membrane raft	6
filamentous actin	3
apical plasma membrane	7
aggresome	3
COP9 signalosome	3
lamin filament	2
proton-transporting two-sector ATPase complex	2
cell	4
cell-cell junction	5
intercalated disc	3
endoplasmic reticulum	12

structural constituent of muscle	8
glycoprotein binding	8
identical protein binding	21
antioxidant activity	5
calcium ion binding	19
serine-type endopeptidase activity	11
actin binding	11
cadherin binding involved in cell-cell adhesion	11
MHC class II protein complex binding	4
protein binding	92
poly(A) RNA binding	20
unfolded protein binding	6
virion binding	3
endopeptidase inhibitor activity	4
actin filament binding	6

cytoskeletal protein binding	4
serine-type endopeptidase inhibitor activity	5
aldehyde dehydrogenase (NAD) activity	3
oxidoreductase activity, acting on the aldehyde or oxo group of donors, NAD or NADP as accepto	3
ATP binding	21
lipoprotein lipase activator activity	2
cysteine-type endopeptidase inhibitor activity	3
aminobutyraldehyde dehydrogenase activity	2
procollagen-proline 4-dioxygenase activity	2
4-trimethylammoniobutyraldehyde dehydrogenase activity	2
Toll-like receptor 4 binding	2
arachidonic acid binding	2
hormone activity	4
peroxiredoxin activity	2

Biosynthesis of amino acids	7
Hypertrophic cardiomyopathy (HCM)	7
Dilated cardiomyopathy	7
Biosynthesis of antibiotics	10
Focal adhesion	9
Protein processing in endoplasmic reticulum	8
Glycolysis / Gluconeogenesis	5
Complement and coagulation cascades	5
Carbon metabolism	6
Cardiac muscle contraction	5
Metabolic pathways	24
Collecting duct acid secretion	3

%	PValue	Genes	d Enrichm	Bonferroni
7.194245	1.64E-11	ACTA1, M	32.02136	1.8E-08
9.352518	4.34E-11	PROS1, PI	15.35782	4.77E-08
9.352518	6.84E-11	MYOM1, '	14.78369	7.53E-08
5.035971	2.26E-05	PRDX4, G	12.16812	0.024523
10.79137	5.97E-05	CTSA, FC	3.650435	0.063536
5.755396	6.01E-05	CAST, SE	8.045035	0.063997
7.913669	0.000197	ALAD, CS	4.402937	0.195134
3.597122	0.000204	ALAD, CF	16.90016	0.200985
5.035971	0.000219	ALAD, AC	8.112077	0.214485
2.877698	0.000225	HSPA8, H	32.44831	0.219255
3.597122	0.000309	TF, PIP, A	15.21014	0.287842
3.597122	0.000373	HSPA8, H	14.48585	0.336715
7.194245	0.000386	CNN2, YV	4.49008	0.345856
3.597122	0.000487	ACTC1, M	13.52013	0.41509
3.597122	0.000787	GPX1, CA	11.92953	0.579489
2.877698	0.000948	HSP90AA	20.28019	0.647881
2.877698	0.001204	PFKFB1, I	18.72018	0.734229
2.877698	0.001346	HRG, S10(	18.02684	0.772821
2.158273	0.001792	ALAD, GI	45.63043	0.860904
2.877698	0.001836	ALDH1L1	16.22415	0.867503
2.158273	0.002291	COPS4, C(	40.56039	0.919804
5.035971	0.002343	IGFBP1, S	5.162231	0.924237
2.158273	0.002849	PFKFB1, /	36.50435	0.956637
2.877698	0.002873	GPX1, GK	13.90642	0.957768
2.877698	0.002873	ATP6V1A	13.90642	0.957768
5.035971	0.002881	ALAD, CS	4.95214	0.958168
3.597122	0.002987	PLA2G2A	8.334326	0.962755
2.158273	0.003463	BHMT, AI	33.18577	0.977989
2.877698	0.003917	GPX1, CA	12.48012	0.986667
2.877698	0.004515	PLEK, HS	11.87133	0.993114
2.158273	0.004859	GPX1, GD	28.08027	0.995291
2.158273	0.004859	HSP90AA	28.08027	0.995291
2.877698	0.005166	ALAD, M'	11.31918	0.996645
5.035971	0.005446	COL18A1,	4.345756	0.997539
2.877698	0.005511	PFKFB1, I	11.06192	0.99771
2.158273	0.005639	F12, APOI	26.07453	0.998012
2.158273	0.005639	TPM1, TN	26.07453	0.998012
2.158273	0.005639	HSPA8, H	26.07453	0.998012
3.597122	0.006063	TAGLN, L	6.83602	0.998756
2.158273	0.008294	GPX1, LM	21.47315	0.999895
2.158273	0.009281	F12, APOI	20.28019	0.999965
2.158273	0.011404	GPX1, CA	18.25217	0.999997
7.194245	0.012353	ACTC1, H	2.674311	0.999999
2.158273	0.012537	F12, PROS	17.38302	0.999999
2.877698	0.012958	CAT, MA'	8.112077	0.999999
2.877698	0.014154	SERPINF1	7.850397	1

2.158273	0.014945	COPS4, C(	15.87146	1
3.597122	0.015916	IGFBP1, C	5.155981	1
2.877698	0.016062	HSP90AA	7.488071	1
2.877698	0.016062	ALAD, PF	7.488071	1
1.438849	0.016251	S100A9, S	121.6812	1
2.877698	0.01741	PFKFB1, I	7.264547	1
2.158273	0.017534	ALAD, CA	14.60174	1
5.035971	0.020578	CNN2, CR	3.251023	1
2.877698	0.021049	TPM4, TP	6.760064	1
2.877698	0.023418	UBQLN1,	6.489662	1
3.597122	0.023547	IRAK2, CA	4.57448	1
1.438849	0.024278	UBQLN1,	81.12077	1
1.438849	0.024278	HSPA8, H	81.12077	1
1.438849	0.024278	S100A9, S	81.12077	1
2.877698	0.025074	PRDX4, G	6.321099	1
2.158273	0.02633	DES, TPM	11.7756	1
2.877698	0.027674	CNN2, TP	6.084058	1
2.158273	0.027937	ATP6V1A	11.40761	1
3.597122	0.031076	BPIFA2, H	4.195902	1
2.158273	0.031267	PFKFB1, I	10.73657	1
2.877698	0.031355	IGFBP1, H	5.794341	1
1.438849	0.03224	S100A9, S	60.84058	1
1.438849	0.03224	F12, F11	60.84058	1
1.438849	0.03224	S100A9, S	60.84058	1
1.438849	0.03224	GPX1, IGI	60.84058	1
2.877698	0.032314	CRP, FCN	5.726172	1
2.877698	0.033288	TPM4, TP	5.659589	1
2.158273	0.038366	LGALS3,	9.606407	1
2.877698	0.038384	CST3, IGF	5.348622	1
1.438849	0.040138	APOH, IG	48.67246	1
1.438849	0.040138	HSP90AA	48.67246	1
1.438849	0.040138	ACTA1, A	48.67246	1
1.438849	0.040138	F12, HPN	48.67246	1
1.438849	0.040138	ACTA1, A	48.67246	1
1.438849	0.040138	CPS1, ASI	48.67246	1
2.158273	0.040228	CRP, HP, I	9.360089	1
1.438849	0.047971	TF, B2M	40.56039	1
1.438849	0.047971	LGALS3,	40.56039	1

68.34532	2.43E-44	YWHAE, (	4.430897	5.55E-42
37.41007	2.77E-23	CRISP1, C	5.06133	6.31E-21
35.97122	2.68E-18	CRISP1, C	4.071674	6.12E-16
49.64029	6.13E-17	YWHAE,	2.728943	2.53E-14
16.54676	6.09E-16	COL18A1,	10.18744	1.27E-13
10.07194	1.36E-10	FCN2, HS	12.07573	3.1E-08
12.23022	4.59E-08	YWHAE,	5.700344	1.05E-05
4.316547	9.46E-08	MYLPF, M	49.16547	2.16E-05
27.33813	1.8E-06	YWHAE,	2.264591	0.000411

4.316547	1.08E-05	ACTA1, M	20.17045	0.002468
5.755396	1.17E-05	YWHAE,	10.38479	0.002654
4.316547	5.43E-05	CNN2, AC	14.56755	0.012315
4.316547	5.94E-05	PROS1, A	14.30268	0.013457
4.316547	0.000133	ACTA1, A	12.10227	0.029826
7.913669	0.000178	CNN2, YV	4.464975	0.039713
2.877698	0.000221	MYH1, M	32.77698	0.04922
5.035971	0.000278	DES, HSP	7.777588	0.061457
4.316547	0.000333	CST3, CO	9.957563	0.073112
2.158273	0.000338	TPM4, TP	98.33094	0.074221
5.755396	0.000655	COL18A1,	5.46283	0.138786
2.877698	0.000865	MYH1, RI	20.97727	0.179036
5.035971	0.001062	ATP6V1A	6.037864	0.215132
3.597122	0.003968	CTSA, HS	7.71223	0.596081
2.158273	0.004881	APOH, SP	28.09455	0.672243
2.158273	0.006372	HSP90AA	24.58273	0.767197
8.633094	0.007649	CST3, HSI	2.533486	0.826329
2.158273	0.012979	MYOM1,	17.10103	0.949132
1.438849	0.015088	CTSA, HS	131.1079	0.968767
2.158273	0.01892	MYH2, M	14.04728	0.987159
3.597122	0.019923	HSPA8, R	4.820144	0.989831
4.316547	0.020353	RAP1B, PI	3.818677	0.990799
2.158273	0.022934	TPM4, TP	12.68786	0.994958
5.035971	0.023518	ATP6V1A	3.153799	0.9956
2.158273	0.025788	UBQLN1,	11.9189	0.997412
2.158273	0.028777	COPS4, C	11.23782	0.998715
1.438849	0.02995	LMNA, LI	65.55396	0.999025
1.438849	0.037297	ATP6V1A	52.44317	0.999828
2.877698	0.040379	PRDX4, P	5.244317	0.999917
3.597122	0.041893	CNN2, RA	3.811277	0.999942
2.158273	0.045602	MYH1, DI	8.740528	0.999976
8.633094	0.049917	CTSA, CA	1.900115	0.999991

5.755396	4.23E-08	MYLPF, M	23.30021	1.39E-05
5.755396	9.35E-07	CTSA, HS	15.05552	0.000307
15.10791	2.64E-06	MYOM1,	3.429703	0.000865
3.597122	1.82E-05	PRDX4, A	30.58152	0.005947
13.66906	2.01E-05	CRP, TPM	3.241556	0.006575
7.913669	4.43E-05	FCN2, PR	5.276812	0.014429
7.913669	9.11E-05	CNN2, MY	4.840241	0.02945
7.913669	0.000129	CNN2, YV	4.639955	0.041426
2.877698	0.000271	YWHAE,	30.58152	0.085072
66.18705	0.000435	YWHAE,	1.281047	0.132941
14.38849	0.001932	YWHAE,	2.166981	0.469663
4.316547	0.002026	HSPA8, H	6.672332	0.485838
2.158273	0.002819	CRP, PPIA	36.69783	0.603901
2.877698	0.004148	CAST, CS	12.23261	0.744243
4.316547	0.004446	TPM4, TP	5.560277	0.76814

2.877698	0.006928	DES, TPM	10.19384	0.897759
3.597122	0.008039	SERPINF1	6.305468	0.929164
2.158273	0.00821	ALDH1L1	21.58696	0.933067
2.158273	0.010214	ALDH1L1	19.31465	0.965526
15.10791	0.017885	ATP6V1A	1.718293	0.997313
1.438849	0.024151	APOC2, A	81.55072	0.999671
2.158273	0.030966	CAST, CS	10.79348	0.999967
1.438849	0.032072	ALDH1L1	61.16304	0.999977
1.438849	0.032072	P4HA2, P4	61.16304	0.999977
1.438849	0.032072	ALDH1L1	61.16304	0.999977
1.438849	0.032072	S100A9, S	61.16304	0.999977
1.438849	0.03993	S100A9, S	48.93043	0.999998
2.877698	0.041081	TG, IGF1,	5.205365	0.999999
1.438849	0.047723	PRDX4, P	40.77536	1
5.035971	0.000243	PKM, CPS	7.776647	0.03503
5.035971	0.000376	ACTC1, D	7.178444	0.053767
5.035971	0.000561	ACTC1, D	6.665698	0.079153
7.194245	0.001142	ACLY, UC	3.773036	0.154674
6.47482	0.003762	RAP1B, M	3.494638	0.425396
5.755396	0.004712	HSPA8, H	3.786432	0.50057
3.597122	0.009218	PKM, PGK	5.969281	0.743696
3.597122	0.010206	F12, PROS	5.796259	0.778647
4.316547	0.012667	PKM, CPS	4.24717	0.846492
3.597122	0.013573	ACTC1, T	5.332558	0.865861
17.26619	0.020589	ATP6V1A	1.574833	0.953024
2.158273	0.043379	ATP6V1A	8.887597	0.998525
