# Supplementary material for: Exploring the Mechanism of Edaravone for Oxidative Stress in Rats with Cerebral Infarction Based on Quantitative Proteomics Technology
Source: Evid Based Complement Alternat Med. 2022 Jan 4;2022:8653697. doi: 10.1155/2022/8653697 (PMC8752268; doi:10.1155/2022/8653697)
Supplement: Supplementary Materials — Table S1: edaravone potential targets and CI genes. Table S2: enrichment analysis results of proteomics. Table S3: enrichment analysis results of edaravone-CI PPI network [file 8653697.f1.zip › 8653697.f1/Table S3.pdf]

Table S3 Enrichment ana

Category	Term
	GO:000759
	GO:003016
	GO:000650
	GO:000257
	GO:004273
	GO:000166
	GO:200035
	GO:001062
	GO:003163
	GO:004306
	GO:005191
	GO:005090
	GO:003019
	GO:007037
	GO:004576
	GO:000695
	GO:004426
	GO:003019
	GO:004866
	GO:000193
	GO:000269
	GO:003057
	GO:001406
	GO:004542
	GO:001054
	GO:003019
	GO:005072
	GO:003411
	GO:003437
	GO:004340
	GO:004320
**BP**	GO:004590
	GO:000197
	GO:001062
	GO:001575
	GO:004353
	GO:007173
	GO:000821
	GO:004340
	GO:000820
	GO:004215
	GO:003052
	GO:001074
	GO:009002

	GO:007126
	GO:190121
	GO:007237
	GO:004215
	GO:000759
	GO:000828
	GO:199031
	GO:004212
	GO:007145
	GO:004590
	GO:000193
	GO:009027
	GO:200037
	GO:004632
	GO:005138
	GO:000600
	GO:190002
	GO:000756
	GO:000152
	GO:000561
	GO:000557
	GO:003109
	GO:000998
	GO:003101
	GO:007006
	GO:000588
	GO:007256
	GO:000989
	GO:000582
**CC**	GO:000557
	GO:000578
	GO:003436
	GO:003109
	GO:007168
	GO:190356
	GO:004302
	GO:000557
	GO:000576
	GO:004512
	GO:004262
	GO:000425
	GO:000202
	GO:000551
	GO:000820

	GO:000194
	GO:000510
	GO:000814
	GO:000487
	GO:001989
	GO:000422
**MF**	GO:000823
	GO:000370
	GO:000550
	GO:000554
	GO:000417
	GO:000406
	GO:000467
	GO:004280
	GO:000551
	GO:004280
	GO:000154
	hsa04610
	hsa04611
	hsa04668
	hsa04931
	hsa04066
	hsa04068
	hsa04621
**PY**	hsa04151
	hsa04915
	hsa04920
	hsa04071
	hsa04722
	hsa04024
	hsa04370

lysis Results of Edaravone-CI PPI network

Description	Count
blood coagulation	20
platelet activation	14
proteolysis	24
platelet degranulation	13
fibrinolysis	8
response to hypoxia	14
negative regulation of endothelial cell apoptotic process	8
positive regulation of gene expression	16
plasminogen activation	6
negative regulation of apoptotic process	20
negative regulation of fibrinolysis	6
leukocyte migration	11
positive regulation of blood coagulation	6
positive regulation of ERK1 and ERK2 cascade	12
positive regulation of angiogenesis	10
inflammatory response	16
cellular protein metabolic process	10
negative regulation of blood coagulation	5
positive regulation of smooth muscle cell proliferation	7
positive regulation of protein phosphorylation	9
positive regulation of leukocyte chemotaxis	5
collagen catabolic process	7
positive regulation of phosphatidylinositol 3-kinase signaling	7
positive regulation of nitric oxide biosynthetic process	6
negative regulation of platelet activation	4
extracellular matrix organization	10
negative regulation of inflammatory response	7
positive regulation of heterotypic cell-cell adhesion	4
low-density lipoprotein particle remodeling	4
steroid hormone mediated signaling pathway	6
response to amino acid	5
positive regulation of vasoconstriction	5
blood vessel remodeling	5
negative regulation of gene expression	8
glucose transport	5
negative regulation of blood vessel endothelial cell migration	4
cellular response to nitric oxide	4
regulation of blood pressure	6
negative regulation of MAP kinase activity	5
cholesterol metabolic process	6
lipoprotein metabolic process	5
intracellular receptor signaling pathway	5
positive regulation of macrophage derived foam cell differentiation	4
positive regulation of monocyte chemotaxis	4

cellular response to mechanical stimulus	6
negative regulation of neuron death	5
blood coagulation, fibrin clot formation	3
lipoprotein catabolic process	3
blood coagulation, intrinsic pathway	4
positive regulation of cell proliferation	13
cellular response to insulin-like growth factor stimulus	3
regulation of cell proliferation	8
cellular response to hypoxia	6
positive regulation of vasodilation	4
negative regulation of endothelial cell proliferation	4
positive regulation of peptide hormone secretion	3
positive regulation of reactive oxygen species metabolic process	4
positive regulation of glucose import	4
response to glucocorticoid	5
glucose metabolic process	5
positive regulation of substrate adhesion-dependent cell spreading	4
aging	7
angiogenesis	8
extracellular space	54
extracellular region	56
platelet alpha granule lumen	11
cell surface	24
extracellular matrix	17
extracellular exosome	51
plasma membrane	62
blood microparticle	11
external side of plasma membrane	11
cytosol	46
fibrinogen complex	4
endoplasmic reticulum lumen	9
low-density lipoprotein particle	4
platelet alpha granule	4
endocytic vesicle lumen	4
extracellular vesicle	5
neuronal cell body	10
proteinaceous extracellular matrix	9
lysosome	8
membrane raft	7
chylomicron	3
serine-type endopeptidase activity	19
protease binding	10
protein binding	101
heparin binding	11

glycoprotein binding	8
receptor binding	15
drug binding	8
RNA polymerase II transcription factor activity, ligand-activated sequence-specific DNA binding	6
enzyme binding	13
metalloendopeptidase activity	8
metallopeptidase activity	7
steroid hormone receptor activity	6
calcium ion binding	17
phospholipid binding	6
endopeptidase activity	5
arylesterase activity	3
protein serine/threonine kinase activity	11
identical protein binding	16
collagen binding	5
protein homodimerization activity	15
beta-amyloid binding	4
Complement and coagulation cascades	18
Platelet activation	12
TNF signaling pathway	10
Insulin resistance	10
HIF-1 signaling pathway	8
FoxO signaling pathway	9
NOD-like receptor signaling pathway	6
PI3K-Akt signaling pathway	14
Estrogen signaling pathway	7
Adipocytokine signaling pathway	6
Sphingolipid signaling pathway	7
Neurotrophin signaling pathway	7
cAMP signaling pathway	9
VEGF signaling pathway	5

%	PValue	Genes	d Enrichm	Bonferroni
14.08451	5.99E-16	FGB, P2R	13.22621	9.86E-13
9.859155	9.58E-12	FGB, FGA	14.81336	1.7E-08
16.90141	1.57E-11	MMP1, F1	5.840696	2.79E-08
9.15493	4.34E-11	FGB, FGA	15.35782	7.71E-08
5.633803	2.18E-10	FGB, FGA	46.35473	3.87E-07
9.859155	1.54E-09	ACVRL1,	9.90428	2.74E-06
5.633803	2.12E-09	FGB, FGA	34.76605	3.76E-06
11.26761	3.77E-09	CRP, TGF	7.43091	6.7E-06
4.225352	4.12E-09	FGB, FGA	81.12077	7.33E-06
14.08451	5.35E-09	GSK3B, G	5.348622	9.5E-06
4.225352	8.19E-09	THBD, AF	73.0087	1.46E-05
7.746479	5.96E-08	SELP, THI	10.97125	0.000106
4.225352	6.34E-08	F7, TBXA	52.14907	0.000113
8.450704	1.95E-07	FGB, FGA	8.343851	0.000347
7.042254	4.16E-07	ACVRL1,	10.58097	0.000739
11.26761	4.86E-07	CRP, EPH	5.136935	0.000863
7.042254	5.18E-07	BACE1, C	10.31196	0.000921
3.521127	2E-06	THBD, PR	50.70048	0.003539
4.929577	9.21E-06	NOTCH3,	14.19614	0.016238
6.338028	9.48E-06	ADAM17,	8.623074	0.01671
3.521127	1.19E-05	F7, IL6, A	33.80032	0.020878
4.929577	1.34E-05	MMP13, C	13.30888	0.023589
4.929577	1.47E-05	SELP, TGI	13.10412	0.025786
4.225352	2.52E-05	IL6, HSP9	16.97877	0.043806
2.816901	2.89E-05	THBD, NC	60.84058	0.050018
7.042254	3.31E-05	FGB, FGA	6.208222	0.05718
4.929577	4.51E-05	PROC, PT	10.78187	0.076956
2.816901	8.36E-05	FGB, FGA	44.24769	0.137998
2.816901	8.36E-05	AGTR1, A	44.24769	0.137998
4.225352	0.0001	RXRA, NI	12.80854	0.162911
3.521127	0.000112	IL6, CASP	19.62599	0.181123
3.521127	0.000128	FGB, FGA	19.01268	0.203024
3.521127	0.000128	ACVRL1,	19.01268	0.203024
5.633803	0.000131	ACVRL1,	7.105469	0.208269
3.521127	0.000144	SLC2A10,	18.43654	0.226328
2.816901	0.000181	ACVRL1,	34.76605	0.275143
2.816901	0.000181	CCNA2, C	34.76605	0.275143
4.225352	0.000187	ACVRL1,	11.23211	0.283214
3.521127	0.000204	PTPN1, G	16.90016	0.304044
4.225352	0.000232	RXRA, PC	10.73657	0.337741
3.521127	0.000252	APOE, AP	16.01068	0.361433
3.521127	0.000252	AR, NR1I	16.01068	0.361433
2.816901	0.000275	PRKCH, A	30.42029	0.386874
2.816901	0.000275	SERPINE1	30.42029	0.386874

4.225352	0.000284	MAPK8, C	10.28291	0.396358
3.521127	0.000309	IL6, CREE	15.21014	0.422112
2.112676	0.000392	FGB, FGA	91.26087	0.502006
2.112676	0.000392	APOE, AP	91.26087	0.502006
2.816901	0.000396	VWF, APC	27.04026	0.505589
9.15493	0.000438	TGFB2, F2	3.394539	0.540938
2.112676	0.00065	CCNA2, C	73.0087	0.685218
5.633803	0.000812	LCK, CHE	5.261888	0.763836
4.225352	0.001133	CCNA2, P	7.605072	0.86672
2.816901	0.001662	NOS3, EPI	16.78361	0.947968
2.816901	0.001662	ACVRL1,	16.78361	0.947968
2.112676	0.001792	FGB, FGA	45.63043	0.958689
2.816901	0.001836	AGTR1, F	16.22415	0.961809
2.816901	0.001836	SLC1A2, A	16.22415	0.961809
3.521127	0.001953	IL6, CASP	9.360089	0.969018
3.521127	0.002184	PIK3CA, A	9.080684	0.979447
2.816901	0.002216	FGB, FGA	15.21014	0.980601
4.929577	0.002343	IL6, CREE	5.162231	0.984519
5.633803	0.002373	ACVRL1,	4.365243	0.985337
38.02817	2.97E-25	HEXB, SE	5.294083	6.86E-23
39.43662	2.49E-23	SERPINE1	4.593321	5.75E-21
7.746479	9.07E-12	FGB, FGA	26.41159	2.1E-09
16.90141	1.58E-11	FGB, P2R	5.847585	3.64E-09
11.97183	7.93E-10	TGFB2, H	7.58441	1.83E-07
35.91549	1.14E-09	HEXB, SE	2.395929	2.64E-07
43.66197	1E-08	ACVRL1,	1.986798	2.32E-06
7.746479	2.24E-07	FGB, FGA	9.556827	5.18E-05
7.746479	4.86E-06	SELP, FGI	6.819895	0.001122
32.39437	2.59E-05	GSK3B, N	1.832479	0.005968
2.816901	3.38E-05	FGB, FGA	58.69243	0.007772
6.338028	0.000103	BACE1, F	6.190217	0.023502
2.816901	0.000142	APOE, AP	37.73085	0.032358
2.816901	0.000142	FGB, FGA	37.73085	0.032358
2.816901	0.000217	HSP90AA	33.01449	0.048819
3.521127	0.000538	FGB, FGA	13.2058	0.116974
7.042254	0.000636	ACVRL1,	4.192317	0.136748
6.338028	0.000962	MMP13, A	4.434783	0.199348
5.633803	0.001619	CST3, HC	4.674618	0.312196
4.929577	0.004684	BACE1, D	4.487407	0.661927
2.112676	0.004812	APOH, AF	28.29814	0.671832
13.38028	2.61E-12	MMP1, F1	9.114493	1.11E-09
7.042254	1.3E-07	DPP4, CS	12.11149	5.55E-05
71.12676	3.88E-07	SERPINE1	1.406367	0.000165
7.746479	7.21E-07	SELP, SEI	8.409918	0.000307

5.633803	9.35E-07	SELP, F7,	15.05552	0.000398
10.56338	1.1E-06	FGB, FGA	5.197992	0.000467
5.633803	2.73E-06	ACE, GST	12.87643	0.001161
4.225352	1.01E-05	AR, RXR	20.38768	0.004283
9.15493	1.75E-05	PTPN1, N	4.775493	0.007408
5.633803	3.75E-05	ADAM17,	8.660254	0.015868
4.929577	5.04E-05	ADAM17,	10.57139	0.021254
4.225352	8.96E-05	AR, RXR	13.10637	0.037471
11.97183	0.000234	CRP, NOT	2.90034	0.094778
4.225352	0.000673	APOH, PC	8.534378	0.249333
3.521127	0.000959	ACE, MM	11.32649	0.335437
2.112676	0.00096	CA2, PON	61.16304	0.335809
7.746479	0.000998	ACVRL1,	3.578689	0.346363
11.26761	0.001122	HSP90AA	2.613107	0.380113
3.521127	0.001424	MMP13, V	10.19384	0.45503
10.56338	0.002453	TGFB2, H	2.51355	0.64871
2.816901	0.002603	BACE1, C	14.3913	0.670565

12.67606	7.44E-17	FGB, FGA	17.25502	2.31E-14
8.450704	3.27E-06	FGB, P2R	6.105621	0.000681
7.042254	2.83E-05	MAPK10,	6.181704	0.005861
7.042254	3.05E-05	MAPK10,	6.124466	0.006314
5.633803	0.00055	IL6, PIK3	5.512019	0.108198
6.338028	0.000846	MAPK10,	4.442523	0.161348
4.225352	0.001423	MAPK10,	7.086882	0.256404
9.859155	0.001803	GSK3B, H	2.684114	0.312999
4.929577	0.003546	HSP90AA	4.676865	0.522327
4.225352	0.003824	MAPK10,	5.669505	0.549293
4.929577	0.008992	MAPK10,	3.858413	0.847218
4.929577	0.008992	MAPK10,	3.858413	0.847218
6.338028	0.009408	MAPK10,	3.006556	0.860016
3.521127	0.01288	PIK3CA,	5.421658	0.93256
